# Kinetics of Precipitation Processes at Non-Zero Input Fluxes of Segregating Particles

**DOI:** 10.3390/e25020329

**Published:** 2023-02-10

**Authors:** Jürn W. P. Schmelzer, Timur V. Tropin, Alexander S. Abyzov

**Affiliations:** 1 Institut für Physik der Universität Rostock, Albert-Einstein-Strasse 23-25, 18059 Rostock, Germany; 2 Competence Centre CALOR, Faculty of Interdisciplinary Research, University of Rostock, Albert-Einstein-Str. 25, 18051 Rostock, Germany; 3 Frank Laboratory of Neutron Physics, Joint Institute for Nuclear Research, ul. Joliot-Curie 6, 141980 Dubna, Russia; 4 National Science Center Kharkov Institute of Physics and Technology, Akademicheskaya Street 1, 61108 Kharkov, Ukraine

**Keywords:** nucleation, thermodynamics of nucleation, general theory of phase transitions, theory and models of crystal growth, 64.60.Bd General theory of phase transitions, 64.60.Q- Nucleation, 81.10.Aj Theory and models of crystal growth, 82.60.Nh Thermodynamics of nucleation in physical chemistry and chemical physics

## Abstract

We consider the process of formation and growth of clusters of a new phase in segregation processes in solid or liquid solutions in an open system when segregating particles are added continuously to it with a given rate of input fluxes, Φ. As shown here, the value of the input flux significantly affects the number of supercritical clusters formed, their growth kinetics, and, in particular, the coarsening behavior in the late stages of the process. The detailed specification of the respective dependencies is the aim of the present analysis, which combines numerical computations with an analytical treatment of the obtained results. In particular, a treatment of the coarsening kinetics is developed, allowing a description of the development of the number of clusters and their average sizes in the late stages of the segregation processes in open systems, which goes beyond the scope of the classical Lifshitz, Slezov and Wagner theory. As is also shown, in its basic ingredients, this approach supplies us with a general tool for the theoretical description of Ostwald ripening in open systems, or systems where the boundary conditions, like temperature or pressure, vary with time. Having this method at one’s disposal supplies us with the possibility that conditions can be theoretically tested, leading to cluster size distributions that are most appropriate for desired applications.

## 1. Introduction

For a number of technological applications in materials science the understanding of the kinetics of phase transformation processes, under varying external and/or internal conditions, is of significant importance. One example in this respect consists of the widely studied process of phase formation at cooling and heating by fast scanning calorimetry [1,2,3,4]. The time-dependence of the supersaturation may be caused also by variations in the concentration of the particles realizing the phase formation, like in condensation processes in expanding gases [5,6], or processes of formation of polymeric foams by bubble nucleation and growth processes of gases dissolved in the liquid [7]. In the course of the phase formation, the state of the ambient phase is generally changed. Such modifications affect the nucleation rate and determine the overall course of the transformation kinetics [8,9,10].

Another similar, but even more complex, circle of investigations consists in the theoretical description of the processes of formation and growth of ensembles of clusters in precipitation processes, when the segregating particles are added homogeneously to the bulk of a solid or liquid solution by a constant or changing in time rate, Φ. In application to photography, the latter problem was analyzed theoretically and experimentally by Leubner [11,12], for both diffusion− and kinetically−limited growth modes of the segregating clusters of the newly evolving precipitating phase. Leubner argued that, for diffusion−limited growth the number of supercritical clusters, *N*, formed as the result of an interplay of nucleation, growth and supply of additional monomers, is proportional to Φ, while for kinetically−limited growth N〈R〉≈constant should hold [12]. Here 〈R〉 is the average size (radius) of the evolving distribution of segregating particles.

However, Leubner’s treatment was based exclusively on the consideration of the mass balance equation interrelating the growth of the clusters with the input fluxes of monomers of the segregating component. An adequate theoretical description of nucleation was lacking. Moreover, a number of important parameters of the theory were not consistently determined in Leubner’s approach. A first analysis of this circle of problems was performed by some of us in [13], and reviewed briefly in [14]. Since the problem discussed by Leubner is of general theoretical and practical interest (see e.g., [15,16,17,18,19]) it is revisited and advanced in the present paper into a comprehensive treatment. As is demonstrated here as well, in its basic ingredients the method advanced supplies us with a general tool for the theoretical treatment of Ostwald ripening in open systems, or systems where the boundary conditions, like temperature or pressure, are changing with time. Consequently, a general tool for the theoretical treatment of coarsening in open systems or systems at time-dependent boundary conditions is here developed, which can be applied to the solution of a variety of problems in materials science and technology.

In the present analysis, we consider the process of formation and growth of clusters of a new phase in segregation processes in solid or liquid solutions. For the case that the initial state is created very fast and that the input fluxes of raw material are equal to zero once this state is reached, the first stages of the kinetics of nucleation and growth of the segregating particles have been analyzed in detail in [20,21,22,23]. Supplemented by the theoretical treatment of the late stages of this process, the stage of coarsening, or Ostwald ripening, first described theoretically by Lifshitz and Slezov [24,25,26], a comprehensive theory of the kinetics of segregation processes in solutions in terms of classical nucleation theory has been advanced in this way. These results and further developments are reviewed and summarized also in the monograph of V. V. Slezov [14].

**Figure 1:**
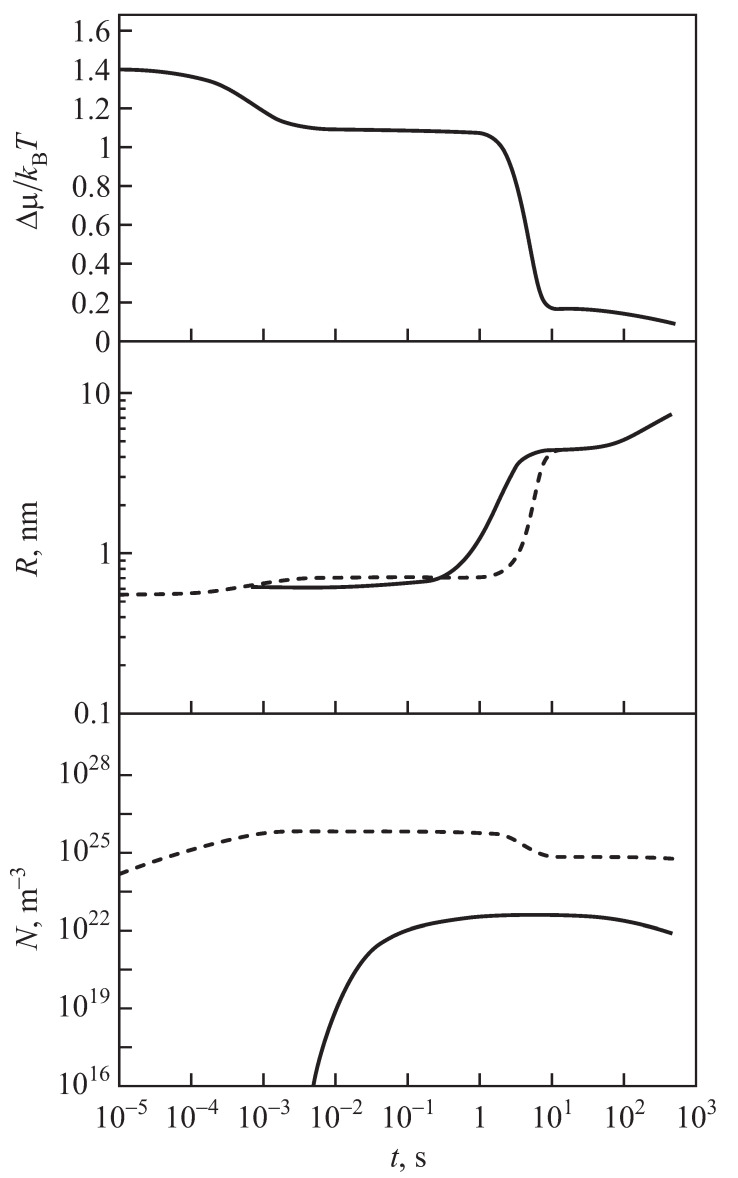
Evolution of characteristic properties of a system undergoing a precipitation process for the case of zero input fluxes of segregating particles. (**Top**): Change of the supersaturation, $\Delta \mu /k_{B}T$, as a function of time (cf. Equation (1)). (**Center**): Change of the average (full curve) and the critical cluster sizes (dotted curve) in the course of the transformation. (**Bottom**): Change of the number of clusters in the system. Herein the dotted curve counts all clusters in the system, while the full curve refers to clusters with a radius R>0.6 nm. In these computations, the aggregation processes were determined for small cluster sizes by kinetically−limited growth, going over continuously to diffusion−limited growth for larger cluster sizes (the figures were taken with permission from [20,21], where further details can also be found).

Typical curves describing the kinetics of segregation for the case of zero input fluxes of segregating particles are shown in Figure 1. As illustrated in this figure, some time (the time−lag in nucleation) is required to establish a steady−state nucleation rate. Steady−state nucleation with a constant nucleation rate can be realized only for a finite time due to depletion effects, i.e, the change of the concentration of segregating particles caused by nucleation and growth. The stage of steady−state nucleation is followed by a stage of evolution, dominated by independent growth of the supercritical clusters, which is, then, followed by a stage of competitive growth or coarsening. These dependencies are shown here for comparison with the respective results obtained for non-zero input fluxes of segregating particles. Going beyond these studies, here we consider the description of nucleation and growth processes in solutions when the number of segregating particles continuously changes due to non-zero input fluxes of raw materials.

We analyze the following model situation: In the initial stage, at the time t=0, the concentration of the segregating particles, c(t), is assumed to be equal to the equilibrium solubility, $c_{eq}$, realizing a stable coexistence of the evolving phase with the initially existing ambient phase at a planar interface. Starting with such an initial state, particles of the segregating phase are homogeneously added, with a constant rate, Φ, to the system. As a result the system is transferred into a metastable state, and the spontaneous formation of supercritical clusters becomes possible (see also [13,14] for more details). The input flux of the segregating phase, Φ, also remains constant when the nucleation and growth processes have started and are proceeding. Its value significantly affects the number of supercritical clusters formed, their growth kinetics, and, in particular, the coarsening behavior at the late stages of the process. The detailed specification of the respective dependencies is the aim of the present analysis.

For these purposes, in Section 2, the basic set of kinetic equations of classical nucleation theory is formulated. Based on its numerical solution, the process of cluster formation and growth at a constant rate of supply of monomers of the segregating component, both for diffusion− and kinetically−limited growth, are analyzed and compared with the case of zero input fluxes of segregating particles. Characteristic quantities, like the average (〈R〉) and critical ($R_{c}$) cluster sizes, and the number of supercritical clusters formed, are discussed. Particular attention is devoted to answering the question as to how the number of stable clusters formed in the system depends on the rate of input fluxes of monomers.

In Section 3, results of numerical computations are compared with theoretical predictions. Three topics are analyzed: (i) Determination of the number of clusters formed in the system in dependence on the input flux of segregating particles; (ii) Analytical description of the late stages of coarsening, in line with the Lifshitz, Slezov and Wagner theory, as an introduction to the theoretical description and to compare with the next item; (iii) Application of an alternative approach [8,27,28] to the description of coarsening and its application to coarsening at non-zero input fluxes of segregating particles. A summary of the results and their discussion completes the paper.

## 2. Precipitation Kinetics at Non-Zero Input Fluxes of Segregating Particles: Numerical Computations

### 2.1. Some General Considerations

Assuming a perfect solution, the relative supersaturation with respect to cluster formation, $\Delta \mu /k_{B}T$, can be expressed as [14,27,28]:

(1) $$\frac{\Delta \mu }{k_{B}T}=\ln\left(\frac{c(t)}{c_{eq}}\right)\;\mathrm{with}\;c\left(t=0\right)=c_{eq}\;.$$

Here Δμ is the difference in the chemical potentials referred to one segregating particle in the ambient and newly evolving phases, respectively, c(t) is the current concentration of segregating particles at time, *t*, and $c_{eq}$ is its equilibrium concentration. $k_{B}$ is the Boltzmann constant and *T* the absolute temperature.

According to classical nucleation theory the probability of formation of stable aggregates of the evolving phase increases with an increasing supersaturation. Consequently, after some period of time, when a sufficiently high value of the concentration of segregating particles is reached, an intensive process of nucleation takes place. Clusters formed in nucleation are stable and capable to further deterministic growth, if they exceed, in size, the actual critical cluster radius, $R_{c}$, given by:

(2) $$R_{c}=\frac{2\sigma }{c_{\alpha }k_{B}T\ln\left(\frac{c(t)}{c_{eq}}\right)}\;.$$

Here $c_{\alpha }$ is the density of segregating particles in the newly evolving phase, while σ denotes the surface tension. In the present analysis, the surface tension is approximately considered to be independent of cluster size, i.e., the capillarity approximation (as it is denoted in classical nucleation theory (CNT)) is employed.

The growth of the clusters in segregation processes in solutions is commonly described via [14,24,25,27,28]:

(3) $$\frac{dR}{dt}=\frac{2\sigma Dc}{c_{\alpha }^{2}k_{B}T}\frac{1}{R}\left(\frac{1}{R_{c}}-\frac{1}{R}\right)$$

for diffusion−limited growth, while for kinetically−limited growth the following relation is employed:

(4) $$\frac{dR}{dt}=\frac{2\sigma Dc}{c_{\alpha }^{2}k_{B}T}\frac{1}{l_{0}}\left(\frac{1}{R_{c}}-\frac{1}{R}\right)\;.$$

Other modes of growth can be modeled by modifications of these relations [14,25].

In Equations (3) and (4), *D* is the diffusion coefficient of the segregating particles in the ambient phase, *R* the radius of the cluster and $l_{0}$ a length parameter with a magnitude of the order of molecular dimensions. We set this parameter as equal to the radius, $R_{1}$, of the segregating particles (monomers), i.e., $l_{0}=R_{1}$.

Processes of formation and growth of clusters of the newly evolving phase result in a sharp reduction of supersaturation, so that, after some interval of time, a steady state might develop, where the formation of new clusters is practically excluded. In this state, the input fluxes of monomers are utilized for the growth of the already existing clusters. Experimental examples for the establishment of such steady states are given by Leubner in his already cited papers [11,12]. In this stage, two limiting mechanisms for the further evolution of the already existing ensemble of clusters can be imagined:

(i) The rate of input fluxes of monomers is small, so the well-known dissolution–growth mechanism of Ostwald ripening dominates, as first described theoretically by Lifshitz and Slezov [14,24,25,26] for the case of zero input fluxes of raw materials. In this case, the behavior of the cluster ensemble is governed by the equations:

(5) $${\langle R\rangle }^{3}∼t\;,\;N∼t^{-1}$$

for diffusion−limited growth (considered in the first papers by Lifshitz and Slezov) and

(6) $${\langle R\rangle }^{2}∼t\;,\;N∼t^{-3/2}$$

for kinetically−limited growth (first analyzed with reference to the Lifshitz–Slezov theory by Wagner [29]). Here *N* is the total number of clusters in the system under consideration in the range of particle numbers in the clusters from $i=i_{min}$ up to $i=i_{max}$ and 〈R〉 is their mean radius given by:

(7) $$\langle R\rangle =\frac{\sum_{i=i_{min}}^{i_{max}}N\left(i,t\right)R}{\sum_{i=i_{min}}^{i_{max}}N\left(i,t\right)}\;.$$

**Figure 2:**
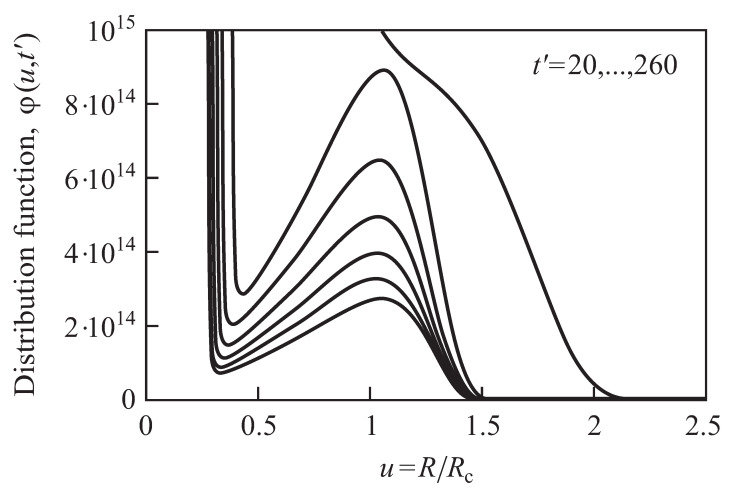
Cluster size distribution function $\varphi (u,t^{\prime })$ in reduced variables $u=R/R_{c}$ for different moments of (dimensionless) time, $t^{\prime }$ (cf. Equation (19)). In the course of the evolution, a time−independent shape of the distribution develops as predicted first by Lifshitz and Slezov (see [14,20,21,24,25] for details). In the numerical computations shown in the figure, the aggregation processes are determined for small cluster sizes via kinetic−limited growth going over continuously to diffusion−limited growth for larger cluster sizes. The figures were taken with permission from [20,21], where further details are also given.

For different purposes, different choices of $i=i_{min}$ turn out to be appropriate. The main reason for such different choices is illustrated in Figure 2. In the figure, the cluster size distribution function $\varphi (u,t^{\prime })$ (here not normalized) is shown in reduced variables $u=R/R_{c}$ for different moments of time. In the course of the evolution, a time-independent shape develops as predicted first by Lifshitz and Slezov (see [14,20,21,24,25,26] for details). In the Lifshitz–Slezov theory, the peak in the distribution in small cluster sizes is not accounted for in computing the average cluster sizes. We similarly proceed here by choosing an appropriate value of $i_{min}$.

(ii) The rate of input fluxes of monomers is sufficiently high to allow an independent simultaneous growth of the already formed supercritical clusters. In this case, we have to expect initially:

(8) $${\langle R\rangle }^{2}∼t\;,\;N∼\mathrm{constant}$$

for diffusion−limited growth and

(9) 〈R〉∼t,N∼constant

for kinetically−limited growth. For closed systems, these conditions are realized in the stage of independent growth if the clusters formed in nucleation are growing mainly at the expense of monomers dissolved in the ambient phase.

(iii) In the course of time, a steady−state is expected to be established in the system, wherein the concentration of the segregating particles in the solution remains nearly constant. The change of the total amount of the precipitating phase has then to be equal to the number of monomers added to the system in the same time interval. This condition yields:

(10) $$\frac{d}{dt}\left[\frac{4\pi }{3v_{\alpha }}{\langle R\rangle }^{3}N\right]=\frac{dN_{1}}{dt}=\Phi \;.$$

Here $v_{\alpha }$ is the volume of a monomer in the evolving phase, $N_{1}$ the total number of monomers. Obviously, neither of the mentioned limiting growth mechanisms, described by Equations (5)–(9), fulfils the restriction given by Equation (10).

It follows that both independent growth, at the expense of additionally introduced monomers, as well as growth−dissolution effects, have to be taken into account to understand the establishment of the steady−state experimentally observed. Consequently, the coarsening behavior is, in general, qualitatively modified by the input fluxes of segregating particles. If, however, the asymptotic behavior is also in this case governed by power laws:

(11) $${\langle R\rangle }^{3}∼t^{\alpha }\;,\;N∼t^{\beta }\;,$$

then, according to Equation (10), the following additional condition holds:

(12) α+β=1.

Which of these conditions is fulfilled is checked, now, by formulating the basic kinetic equations describing segregation processes in solutions and solving them numerically.

### 2.2. Basic Kinetic Equations

In accordance with the classical theory of nucleation and growth we assume that growth and decay processes of clusters proceed only via addition or removal of monomers. The clusters are assumed to be of spherical shape and are characterized by the number of monomers, *i*, contained in them, or by a radius, *R*. The quantity N(i,t) denotes the number of clusters consisting of *i* monomers. The number density in a continuous description is denoted as f(R,t).

The time evolution of the cluster size distribution function, N(i,t), is described, in accordance with these assumptions, by the following set of equations [13,14,20,21,22,23,27,28,30,31,32]:

(13) $$\begin{array}{cc} \frac{\partial N(i,t)}{\partial t} & =w^{\left(+\right)}\left(i-1,t\right)N\left(i-1,t\right)+w^{\left(-\right)}\left(i+1,t\right)N\left(i+1,t\right)\\ \; & -\left[w^{\left(+\right)}\left(i,t\right)+w^{\left(-\right)}\left(i,t\right)\right]N\left(i,t\right)\;,\;i\geq 2\;. \end{array}$$

The number of monomers in the solution, N(1,t), is governed by the mass balance equation:

(14) $$\sum_{i=1}^{\infty }iN\left(i,t\right)=c\left(t=0\right)V+\Phi \;.$$

Here *V* is the volume of the solution.

In order to solve this set of kinetic equations, we have to specify the expressions for the rates of attachment $w^{\left(+\right)}$ and dissolution $w^{\left(-\right)}$ for different deterministic growth mechanisms. For diffusion−limited growth we have (for the limiting case of a perfect solution):

(15) $$\begin{array}{cc} w^{\left(+\right)}\left(i,t\right) & =4\pi RDc=4\pi DcR_{1}i^{1/3}\;, \end{array}$$

while for kinetically−limited growth:

(16) $$w^{\left(+\right)}\left(i,t\right)=4\pi R^{2}D\frac{c}{R_{1}}=4\pi DcR_{1}i^{2/3}$$

holds with:

(17) $$v_{\alpha }=\frac{1}{c_{\alpha }}=\frac{4\pi }{3}R_{1}^{3}\;.$$

The quantity $v_{\alpha }$ denotes the volume of a monomeric unit with a radius, $R_{1}$, in the segregating phase. The density of particles in that phase is then given by $c_{\alpha }$. Taking into account latter relation the radius, *R*, of a cluster may be expressed as:

(18) $$R={\left(\frac{3v_{\alpha }}{4\pi }\right)}^{1/3}i^{1/3}\;.$$

For the numerical calculations, we use a dimensionless time scale defined by:

(19) $$t^{\prime }=4\pi Dc_{eq}{\left(\frac{3v_{\alpha }}{4\pi }\right)}^{1/3}t=4\pi Dc_{eq}R_{1}t\;.$$

Once the attachment coefficients are known, the coefficients of emission can be determined via [14,27,28]:

(20) $$\frac{w^{\left(+\right)}\left(i,t\right)}{w^{\left(-\right)}\left(i+1\right)}=\exp\left\{-\frac{\Delta G(i+1)-\Delta G(i)}{k_{B}T}\right\}\;.$$

Conventionally, these relations are derived employing the principle of detailed balancing. As shown by some of us in cooperation with V. V. Slezov [33], it can be obtained also in an alternative more reliable way not using mentioned principle known to be strictly valid only for thermodynamic equilibrium states.

Employing Equation (20), for diffusion−limited growth we obtain:

(21) $$\begin{array}{cc} \frac{\partial N(i,t^{\prime })}{\partial t^{\prime }} & =\left(\frac{c}{c_{\left(eq\right)}}\right)\{{\left(i-1\right)}^{1/3}N\left(i-1,t^{\prime }\right)+\\ \; & +\left.i^{1/3}N\left(i+1,t^{\prime }\right)\exp\left[\frac{\Delta G(i+1)-\Delta G(i)}{k_{B}T}\right]\right\}-\\ \; & -\left(\frac{c}{c_{\left(eq\right)}}\right)\left\{{\left(i-1\right)}^{1/3}\exp\left[\frac{\Delta G(i)-\Delta G(i-1)}{k_{B}T}\right]+i^{1/3}\right\}N\left(i,t^{\prime }\right)\;. \end{array}$$

Similarly, for kinetic−limited growth we get:

(22) $$\begin{array}{cc} \frac{\partial N(i,t^{\prime })}{\partial t^{\prime }} & =\left(\frac{c}{c_{\left(eq\right)}}\right)\{{\left(i-1\right)}^{2/3}N\left(i-1,t^{\prime }\right)\\ \; & +\left.i^{2/3}N\left(i+1,t^{\prime }\right)\exp\left[\frac{\Delta G(i+1)-\Delta G(i)}{k_{B}T}\right]\right\}\\ \; & -\left(\frac{c}{c_{\left(eq\right)}}\right)\left\{{\left(i-1\right)}^{2/3}\exp\left[\frac{\Delta G(i)-\Delta G(i-1)}{k_{B}T}\right]+i^{2/3}\right\}N\left(i,t^{\prime }\right)\;. \end{array}$$

Here ΔG(i) is given by:

(23) $$\Delta G\left(i\right)=-i\Delta \mu +4\pi \sigma R^{2}\;.$$

### 2.3. Results of the Numerical Solution of the Kinetic Equations

For the numerical solution of Equations (21) and (22), Euler’s Polygon method was used (cf. [30,31]), i.e., the change of the number of clusters consisting of *i* monomers was calculated by:

(24) $$N\left(i,t^{\prime }+\Delta t^{\prime }\right)=N\left(i,t^{\prime }\right)+\frac{\partial N(i,t^{\prime })}{\partial t^{\prime }}\;\Delta t^{\prime }\;.$$

**Figure 3:**
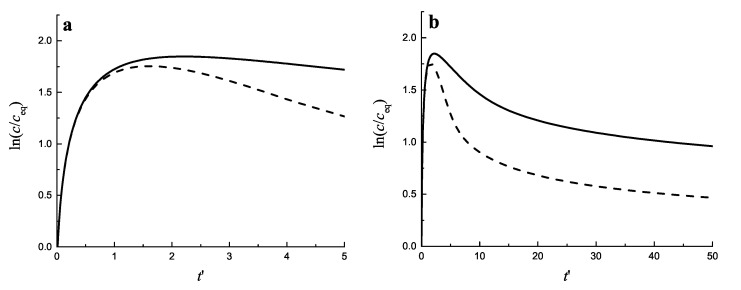
Time-dependence of the supersaturation for diffusion− (full curve) and kinetically−limited growth (dashed curve). (**a**) shows the initial stage of the segregation process, while (**b**) gives an impression of later stages. The values of the parameters were taken from [31], and were set equal to: $c_{eq}\;=\;8.84\cdot 10^{25}\;m^{-3}$, $v_{\alpha }\;=\;4.89\cdot 10^{-29}\;m^{3}$, $R_{1}\;=\;2.269\cdot 10^{-10}\;m$, σ = 0.08N/m, T = 457 °C = 730K, $k_{B}T=10^{-20}\;J$, $D=5.8\cdot 10^{-19}\;m^{2}/s$. With these parameters, Equation (19) yields $t≅6.84t^{\prime }$, and, in a dimensionless time scale, $t^{\prime }$, the input flux of segregating particles is equal to $\Phi =10^{27}\;m^{-3}$.

The values of the parameters, like σ, $c_{\alpha }$ and $c_{eq}$, were taken from reference [31] and are given in the caption to Figure 3.

**Figure 4:**
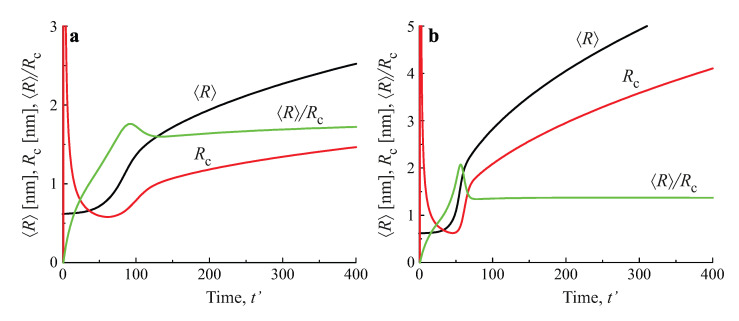
Average cluster radius 〈R〉, critical cluster radius $R_{c}$, and their ratio $\langle R\rangle /R_{c}$ as functions of (dimensionless) time $t^{\prime }$ for (**a**) diffusion−limited growth and (**b**) for kinetically−limited growth. The input flux of segregating particles is equal to $\Phi =10^{25}\;m^{-3}$. The average size and number of clusters is determined, again, in line with Equation (7), taking $i_{min}$ equal to $i_{min}=20$.

In Figure 3 the change of the relative supersaturation $\ln(c/c_{eq})$ is presented, starting from an initial value equal to zero (assuming that $c\left(t=0\right)=c_{eq}$ holds). As seen, initially the supersaturation grows monotonically. After some sufficiently large value of the concentration is reached intensive nucleation occurs. The processes of formation and further growth of the clusters diminish the supersaturation. In Figure 4a,b the time evolution of the average cluster radius 〈R〉, the critical cluster radius $R_{c}$ and their ratio $\langle R\rangle /R_{c}$ is shown both for diffusion−*(a)* and kinetically−limited growth *(b)*.

**Figure 5:**
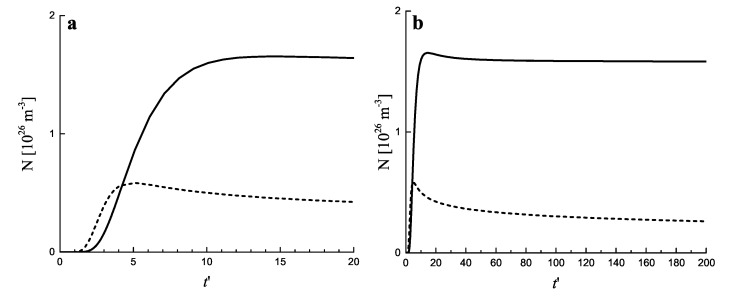
Number of clusters per cubic meter as a function of (dimensionless) time, $t^{\prime }$, for diffusion− (full curve) and kinetically−limited growth (dashed curve). (**a**) shows the initial stage while (**b**) also illustrates the behavior in advanced stages of the process. The input flux of segregating particles is equal to $\Phi =10^{27}$ m ${}^{-3}$. The number of clusters is determined in line with Equation (7) taking $i_{min}$ equal to $i_{min}=20$.

**Figure 6:**
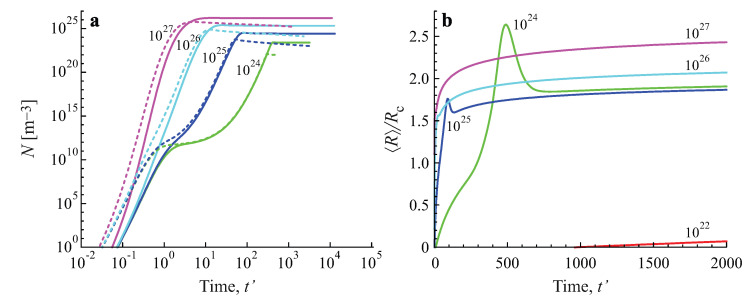
Number of clusters per cubic meter as a function of (dimensionless) time, $t^{\prime }$, for diffusion− (full curves) and kinetically−limited growth (dashed curves) in dependence on the value of the input fluxes, Φ (**a**). The approach to the asymptotic stage can hereby be quite different in dependence on the value of the input flux, as illustrated for diffusion−limited growth in (**b**) plotting the ratio of the average cluster size 〈R〉 and the critical cluster size, $R_{c}$. The average size and number of clusters is determined, again, in line with Equation (7) taking $i_{min}$ equal to $i_{min}=20$.

In Figure 5 the same processes are illustrated by considering the variation of the number of clusters in the system in time. It is seen that for both growth modes a steady state is asymptotically reached, characterized by a practically constant number of clusters. The variation of the number of clusters as a function of time in dependence on the value of the input fluxes of particles is illustrated in Figure 6a. The approach to the asymptotic value of the cluster number can hereby be quite different in dependence on the input fluxes, as illustrated in Figure 6b.

**Figure 7:**
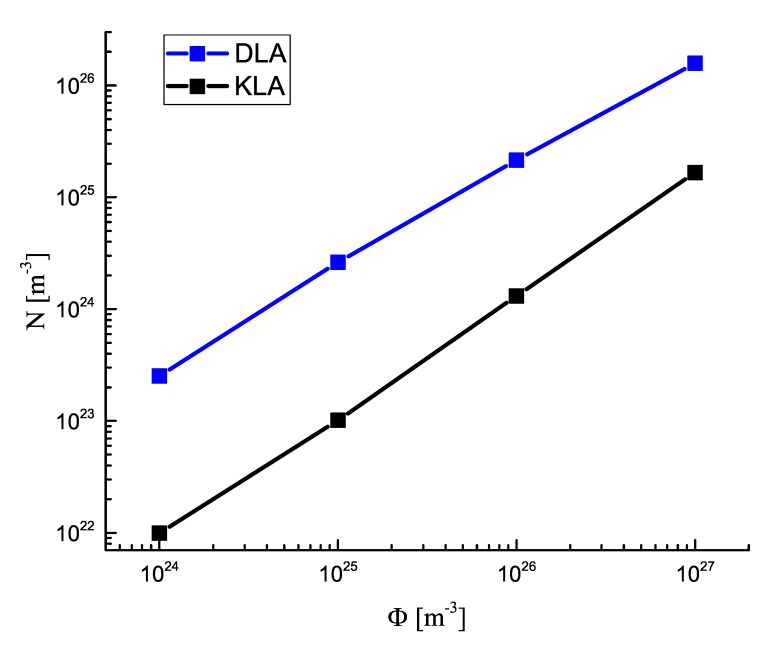
Asymptotic value of the number of clusters per cubic meter for diffusion− and kinetic−limited growth as a function of the input flux of monomers Φ.

In Figure 7, the asymptotic values of the number of supercritical clusters are shown as a function of the input fluxes of monomeric building units, Φ. As is evident, for the considered interval of values of Φ, in agreement with Leubner’s results, a linear dependence *N* vs. Φ was found.

The analysis of the figures further leads to the conclusion that Equation (10) holds in a good approximation. In addition, Equation (11) was also partly fulfilled. For diffusion−limited aggregation, α≅1 and β≅0 hold for all values of the studied input fluxes of segregating particles. For kinetic−limited growth, we obtained α≅1.225 and β≅−0.223 at $\Phi =10^{27}\;m^{-3}$ and α≅1.366 and β≅−0.340 at $\Phi =10^{25}\;m^{-3}$.

## 3. Theoretical Analysis

### 3.1. Number of Clusters in Dependence on the Rate of Input Fluxes of the Segregating Component

The analytical description of nucleation−growth processes at varying state parameters of the ambient phase, where these processes take place, is a highly complex problem (see e.g., [34,35,36,37,38,39,40,41]). In the realization of such task, one has to employ by necessity certain appropriate approximations.

In the present paper, we develop a theoretical estimate of the number of clusters formed by nucleation in dependence on the rate of input fluxes of segregating particles, employing methods described in detail in [35,40]. The strategy can be described as follows: The time of formation of the first supercritical nucleus, or the degree of supersaturation reached at this moment, depends on the rate of supply of segregating particles to the solution. On the other hand, the number of clusters formed in a solution in dependence on supersaturation can be determined as described in [14,20,23]. By identifying the supersaturation with its value reached at the time of formation of the first supercritical nucleus, we can get in this way immediately relations for the dependence of the number of clusters formed in nucleation on the rate of supply of segregating particles.

We consider, in detail, nucleation and growth processes with constant input fluxes of the segregating component. The process is assumed to start at time t=0 with the equilibrium concentration, $c_{eq}$, in the solution. Accounting for Equation (10), we get:

(25) $$c\left(t\right)=c_{eq}+\left(\frac{\Phi }{V}\right)t\;.$$

The steady−state nucleation rate, J(c(t)), as given in terms of classical nucleation theory, is a function of the current concentration. It increases with increasing concentration. For segregation in solutions, we can generally write in terms of CNT:

(26) $$J=J_{0}\exp\left(-\frac{W_{c}}{k_{B}T}\right)\;,\;W_{c}=\frac{16\pi }{3}\frac{\sigma^{3}}{{\left(c_{\alpha }\Delta \mu \right)}^{2}}\;.$$

Employing the model of a perfect solution, we obtain with Equation (1):

(27) $$J=J_{0}\exp\left(-\frac{16\pi }{3}\frac{\sigma^{3}}{{\left(k_{B}T\right)}^{3}{\left(c_{\alpha }\ln\left(\frac{c}{c_{eq}}\right)\right)}^{2}}\right)\;.$$

The pre-exponential term, $J_{0}$, in the expression for the steady−state nucleation rate, reflects the kinetics of the aggregation processes. Its value is determined basically by the diffusion coefficient and the number density of the segregating particles in the ambient phase. It also depends, consequently, on concentration, but this dependence is weak, compared with the exponential term. For this reason, we consider $J_{0}$ as a constant in the subsequent transformation (see e.g., also [34]).

In addition, we assume that the surface tension does not change in dependence on concentration (capillarity approximation). An account of the dependence of the surface tension on concentration, or of the size of the critical clusters, can easily be incorporated into the description employing relations derived in [42,43,44]. Both the exponential term containing the work of critical cluster formation and the pre-exponential term in Equations (26) and (27) are functions of pressure and temperature (for details, see e.g., [45,46]). In particular, such dependence enters the description via the dependence of the equilibrium solubility on these parameters. Pressure and temperature are also kept constant.

Note that the expressions for the nucleation rate are commonly formulated for a unit volume, $V=1\;\;m^{3}$. For this reason, the number of supercritical clusters, N(t), in dependence on time in a volume, *V*, of the solution, is given by:

(28) $$N\left(t\right)=\int_{t=0}^{t}J\left(c\left(t^{\prime }\right)\right)Vdt^{\prime }\;.$$

Since nucleation is a stochastic process, *J* has to be interpreted as the average rate of formation of supercritical nuclei. The average time, 〈τ〉, at which the first supercritical nucleus is formed, can be expressed, consequently, as:

(29) $$V\int_{t=0}^{\langle \tau \rangle }J\left(c\left(t^{\prime }\right)\right)dt^{\prime }=1\;.$$

At constant values of the steady−state nucleation rate, the well-known expression for the average time of formation of the first supercritical nucleus, 〈τ〉=1/(JV), is obtained. If the nucleation rate changes with time, 〈τ〉 can be obtained by numerical integration or by methods described in detail in [47].

Employing a method proposed by Skripov & Koverda [34], we arrive at an approximate analytical solution. For that purpose, we rewrite Equation (29) as:

(30) $$\int_{t=0}^{\langle \tau \rangle }J\left(c\left(t^{\prime }\right)\right)dt^{\prime }=\int_{0}^{J(\langle \tau \rangle )}\frac{dJ}{\left(d\ln J/dt^{\prime }\right)}=\int_{0}^{J(c(\langle \tau \rangle ))}\frac{dJ}{\left(\Phi /V)(d\ln J/dc\right)}=\frac{1}{V}\;.$$

Since Φ is constant and (dlnJ/dc) is a weak function of concentration, we obtain approximately:

(31) $$\frac{\Phi }{V}\frac{d\ln J(c)}{dc}=J\left(c\right)V\;at\;c=c_{N}$$

for the value of the concentration, $c_{N}$, reached when the first supercritical nucleus is formed. According to Equation (25), we get the following relation:

(32) $$\langle \tau \rangle =\frac{c_{N}-c_{eq}}{\left(\Phi /V\right)}$$

for the average time of formation of the first supercritical nucleus. In this relation, $c_{N}$ has to be determined via Equation (31).

Employing the statistical approach to crystal nucleation [40], we could advance also a more detailed description. The density of probability of evolution of the first critical nucleus, ω(τ), can be expressed as:

(33) $$\omega \left(\tau \right)=J\left(\tau \right)V\exp\left(-\int_{0}^{\tau }J\left(\tau^{\prime }\right)Vd\tau^{\prime }\right)\;.$$

Written in dependence on concentration, we obtain:

(34) $$\omega \left(c\right)=\frac{J(c)V}{\left(\Phi /V\right)}\exp\left(-\int_{0}^{\tau }\frac{J(c^{\prime })V}{\Phi }dc^{\prime }\right)\;.$$

This relation can be reformulated as:

(35) $$\omega \left(c\right)=\frac{J(c)V}{\left(\Phi /V\right)}\exp\left(-\int_{0}^{J}\frac{dJ}{\left(\Phi /V)(d\ln J/dc\right)}\right)\;.$$

Since Φ is constant and (dlnJ/dc) is a much weaker function of concentration, as compared to *J*, we may write approximately:

(36) $$\omega \left(c\right)=\frac{J(c)V}{\left(\Phi /V\right)}\exp\left(-\frac{J(c)}{\left(\Phi /V)(d\ln J/dc\right)}\right)\;.$$

The concentration, $c_{max}$, where the density of probability of evolution of the first critical nucleus, ω(c), reaches its maximum, can be expressed, consequently, as:

(37) $${\left\{\left.\frac{\Phi }{V}\frac{d\ln J(c)}{dc}=J\left(c\right)V\right\}\right|}_{c=c_{max}}\;.$$

A comparison of Equations (31) and (37) shows that the average time of formation of the first supercritical nucleus, 〈τ〉, and the concentration, $c_{N}$, reached at that time, as obtained in terms of CNT, corresponds to the concentration, $c_{max}$, at the maximum of the density of probability of evolution of the first critical nucleus in the stochastic approach to nucleation.

As shown in [14,20,23], the number of clusters formed at a given initial concentration, *c*, at zero input−fluxes of segregating particles can be expressed for kinetic−limited growth as:

(38) $$N_{\left(k\right)}\propto cV\exp\left(-\frac{3}{4}\frac{W_{c}}{k_{B}T}\right)\;,$$

and for diffusion−limited growth as:

(39) $$N_{\left(d\right)}\propto cV\exp\left(-\frac{3}{5}\frac{W_{c}}{k_{B}T}\right)\;.$$

Combining Equations (31), (38), and (39), we obtain the following estimates for the dependence of the number of clusters formed in the system on the rate of input fluxes of segregating particles for kinetic−limited growth:

(40) $$\frac{N_{\left(k\right)}}{c_{eq}V}\propto {\left(\frac{\Phi }{V^{2}}\frac{d\ln J(c)}{dc}\right)}^{3/4}\;,$$

and for diffusion−limited growth:

(41) $$\frac{N_{\left(d\right)}}{c_{eq}V}\propto {\left(\frac{\Phi }{V^{2}}\frac{d\ln J(c)}{dc}\right)}^{3/5}\;.$$

These relations are in qualitative agreement with the results of numerical computations given in Figure 7. A quantitative agreement could not be expected since, as noted, Equations (38) and (39) hold for nucleation in closed systems. Consequently, Equations (40) and (41) can be considered to be lower estimates of the number of clusters formed in open systems at the conditions specified above. Employing the results for the time of steady−state nucleation and the nucleation rate at any given value of the concentration, derived in the papers [14,20,23], one can easily estimate corrections to Equations (40) and (41), determining the number of clusters required to consume the additional amount of particles added to the system.

### 3.2. Coarsening in Closed Systems: Lifshitz-Slezov-Wagner−Approach

In the present section we briefly review the Lifshitz, Slezov and Wagner (LSW) theory, in order to specify the directions in which it can be eventually advanced to describe coarsening at non-zero input fluxes of segregating particles.

As is well−known, the LSW−theory describes the asymptotic stages of Ostwald ripening. In the asymptotic stage of Ostwald ripening, the concentration of segregating particles in the ambient phase has values near to its equilibrium concentration. Consequently, in Equations (3) and (4) *c* can be replaced by $c_{eq}$. Employing the dimensionless time scale $t^{\prime }$ (Equation (19)) and the notation:

(42) $$R_{o}^{3}=\frac{\sigma }{2\pi c_{\alpha }^{2}k_{B}TR_{1}}\;,$$

Equations (3) and (4) can be written for diffusion−limited growth in the form:

(43) $$\frac{dR}{dt^{\prime }}=\frac{R_{o}^{3}}{R}\left(\frac{1}{R_{c}}-\frac{1}{R}\right)\;,$$

and for kinetically−limited growth as:

(44) $$\frac{dR}{dt^{\prime }}=\frac{R_{o}^{3}}{R_{1}}\left(\frac{1}{R_{c}}-\frac{1}{R}\right)\;.$$

In terms of the reduced radius, $u=(R/R_{c})$, and the time scale, $\tau_{d}=3\ln\left(R/R_{c}\right)$, these relations, for diffusion−limited growth, adopt the form:

(45) $$\frac{du^{3}}{d\tau_{d}}=\gamma_{d}\left(1-u\right)-u^{3}\;,\;\gamma_{d}={\left({\left(\frac{R_{c}}{R_{o}}\right)}^{2}\frac{d}{dt^{\prime }}\left(\frac{R_{c}}{R_{o}}\right)\right)}^{-1}\;,$$

and, for kinetic limited-growth, with $\tau_{k}=2\ln\left(R/R_{c}\right)$,

(46) $$\frac{du^{2}}{d\tau_{k}}=\gamma_{k}\left(1-u\right)-u^{2}\;,\;\gamma_{k}={\left(\left(\frac{R_{1}}{R_{o}}\right)\left(\frac{R_{c}}{R_{o}}\right)\frac{d}{dt^{\prime }}\left(\frac{R_{c}}{R_{o}}\right)\right)}^{-1}\;.$$

Following the argumentation put forward by Lifshitz and Slezov [14,24,25], we come to the conclusion that, in the asymptotic stage of coarsening, the relations $\gamma_{d}\left(u\right)=27/4$, $u_{0}=3/2$ hold for diffusion−limited growth, while, for kinetic−limited growth, $\gamma_{k}\left(u\right)=4$, $u_{0}=2$ is fulfilled. Here $u\leq u_{0}$ specifies the range where clusters are found in cluster size space. These conditions result in the following dependencies for the critical cluster radii, for diffusion−limited growth:

(47) $${\left(\frac{R_{c}\left(t^{\prime }\right)}{R_{o}}\right)}^{3}={\left(\frac{R_{c}\left(t^{\prime }=0\right)}{R_{o}}\right)}^{3}+\frac{4}{9}t^{\prime }\;,$$

and for kinetic−limited growth:

(48) $${\left(\frac{R_{c}\left(t^{\prime }\right)}{R_{o}}\right)}^{2}={\left(\frac{R_{c}\left(t^{\prime }=0\right)}{R_{o}}\right)}^{2}+\frac{R_{o}}{2R_{1}}t^{\prime }\;.$$

Accounting for the mass balance equation, in the case of zero input fluxes of segregating particles, the number of clusters changes with time as $N\propto 1/t^{\prime }$ for diffusion−limited growth and $N\propto 1/{\left(t^{\prime }\right)}^{3/2}$ for kinetic−limited growth.

The cluster size distribution in reduced units, $u=(R/R_{c})$, can be written as:

(49) $$\varphi \left(u,\tau \right)=N\left(\tau \right)P\left(u\right){\left(\langle R\rangle \right)}^{-1}\;,$$

(50) $$\langle R\rangle =R_{c}\frac{\int_{0}^{u_{0}}P_{n}\left(u\right)udu}{\int_{0}^{u_{0}}P_{n}\left(u\right)du}\;,$$

where P(u), for diffusion−limited growth, takes the form [14,24,25]:

(51) $$P\left(u\right)=\frac{3^{4}e}{2^{5/3}}\frac{u^{2}\exp\left\{-\frac{1}{1-\left(2/3\right)u}\right\}}{{\left(u+3\right)}^{7/3}{\left(\frac{3}{2}-u\right)}^{11/3}}\;,\;u\leq u_{0}=\frac{3}{2}\;,$$

$$P\left(u\right)=0\;,\;u\geq u_{0}=\frac{3}{2}$$

and for kinetic−limited growth:

(52) $$P\left(u\right)=\frac{24e^{3}u\exp\left\{-\frac{6}{2-u}\right\}}{{\left(2-u\right)}^{5}}\;,\;u\leq u_{0}=2\;,$$

$$P\left(u\right)=0\;,\;u\geq u_{0}=2\;.$$

The approach to such time-independent distributions is illustrated in Figure 2.

The results concerning the average cluster size and the number of clusters in dependence on time, obtained numerically and discussed here in connection with Figure 7, cannot be described by the LSW−theory in the form presented. We consider the issue as to whether this approach could be employed in an appropriate modification to the analysis of coarsening at non−zero input fluxes of segregating particles as an open problem. In the present paper, we concentrate the attention on an alternative approach to the description of the kinetics of coarsening described in subsequent sections of the paper.

### 3.3. Coarsening at Constant Input Fluxes of the Segregating Component: Alternative Approach

#### 3.3.1. Basic Ideas

In cooperation with I. S. Gutzow and R. Pascova, in 1984–85, an alternative approach to the description of Ostwald ripening in liquid and solid solutions was advanced by some of us [48,49], based on thermodynamic evolution laws. The method was applied to the description of experimental data on coarsening under the influence of elastic stresses of different types [8,27,28,50,51,52]. A direct confirmation of the basic ideas underlying this approach is given in [53], demonstrating that coarsening can be interpreted as the result of the evolution along a properly chosen “thermodynamic potential valley”. It was further shown in [8,9,10,54,55,56,57,58] that this method was similarly valid for the description of coarsening in a variety of alternative realizations. In particular, in adiabatic systems coarsening proceeded via an appropriately defined valley of the entropy of the heterogeneous systems, consisting of a distribution of clusters of different numbers and sizes in the ambient phase.

The basic idea of this method of theoretical description of coarsening is connected with the replacement of the real ensemble of clusters of the newly evolving phase by an idealized model, consisting of *N* identical clusters with the same size, *R*, which can be identified with the average cluster radius, 〈R〉, of the real ensemble evolving in the ambient phase. Employing this model, the appropriate thermodynamic potential has to be computed in dependence on cluster number, *N*, and average size, 〈R〉. We here consider phase formation at constant values of pressure and temperature, and the appropriate thermodynamic potential is, consequently, the Gibbs free energy, ΔG.

**Figure 8:**
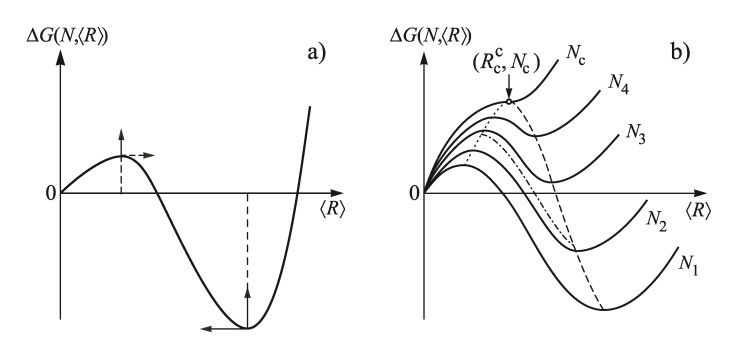
Change of the Gibbs free energy, ΔG(N,〈R〉), in dependence on (average) cluster size, 〈R〉, for different values of the number of clusters, *N* ($N_{1}<N_{2}<\ldots <N_{c}$). In (**a**), ΔG(N,〈R〉) is shown for one given value of the number of clusters. The arrows indicate the change of the position of the extremum values of ΔG(N,〈R〉) with increase of *N*. In (**b**), the respective curves are shown for a set of different values of the number of clusters. The dotted curve describes nucleation, the dashed−dotted curve describes the stage of dominating independent growth, while the dashed curve refers to coarsening. The minimum value of the average cluster size and the maximum number of clusters, which may start to evolve via Ostwald ripening, is denoted by $R_{c}^{c}$, respectively, $N_{c}$.

The typical shape of the function ΔG(N,〈R〉) is shown for constant values of *N* in dependence on 〈R〉 in Figure 8. Employing the results illustrated in this figure, one can develop a general scenario of segregation processes in solutions (and a variety of other realizations of phase formation as mentioned above). When the evolution of the new phase is accompanied by a depletion of the ambient phase, a nucleation stage, with increasing (due to depletion) sizes of the critical clusters and of the work of critical cluster formation, is followed by a stage of independent growth of the supercritical clusters, partly accompanied by formation of additional clusters and, finally, by a third stage of competitive growth or Ostwald ripening.

The initial state of coarsening is determined by the time interval at which steady−state nucleation proceeds at given values of pressure and temperature. It determines the state, along the predominant nucleation path (dotted curve), at which nucleation goes over into dominating independent growth (dashed−dotted) curve. This evolution path determines the initial conditions for coarsening. The cluster-size distributions formed in these initial stages further slowly change when the system passes into coarsening. The latter process proceeds at much larger time scales, so, effectively, the cluster size can be maintained at this specific value by cooling the system. The number of clusters in these particular distributions can be varied by changing pressure and/or temperature prior to nucleation, since the duration of the nucleation stage and its intensity is varied by such modifications of the process conditions.

The stage of Ostwald ripening can be described (in an alternative treatment, as compared to the one developed by V. V. Slezov in cooperation with I. M. Lifshitz [14,24,25,26]) by a simple coupled set of two differential equations for the average cluster size, 〈R〉, and their number, *N*, in the system. Taking into account the possibility of evolution of elastic stresses, caused by the formation of a cluster, this set of equations becomes (for the details, see [8,9,50]):

(53) $$\begin{array}{ccc} \frac{d\langle R\rangle }{dt} & = & \omega \frac{Dc}{c_{\alpha }^{2}k_{B}T}\frac{1}{\langle R\rangle l}\left\{\sigma +\frac{3}{4\pi {\langle R\rangle }^{2}}\left[\Phi^{\left(\varepsilon \right)}-\langle V_{\alpha }\rangle \frac{\partial \Phi^{\left(\varepsilon \right)}}{\partial \langle V_{\alpha }\rangle }\right]\right\}\\ & & \times \frac{1}{Z}\left\{1+Z-\frac{{\langle R\rangle }^{2}}{2\sigma }\frac{\partial^{2}\Phi^{\left(\varepsilon \right)}}{\partial \langle R\rangle \partial \langle V_{\alpha }\rangle }\right\}\;, \end{array}$$

(54) $$\begin{array}{c} \frac{d}{dt}\ln\left(N{\langle R\rangle }^{3}\right)=-\frac{1}{Z}\left\{\left[1-\frac{{\langle R\rangle }^{2}}{2\sigma }\frac{\partial^{2}\Phi^{\left(\varepsilon \right)}}{\partial \langle R\rangle \partial \langle V_{\alpha }\rangle }\right]\right\}\frac{d}{dt}\left[\ln{\langle R\rangle }^{3}\right] \end{array}$$

with

(55) $$l=\left\{\begin{array}{cc} \langle R\rangle & \mathrm{for diffusion–limited growth}\\ \; & \\ l_{0} & \mathrm{for kinetic–limited growth} \end{array}\right.\;.$$

Here *V* is the total volume of the system, $\langle V_{\alpha }\rangle$ is the average volume of the clusters and *Z* is a parameter, which has a value equal to minus one at the state indicated in Figure 8 by the inflection point, ($N_{c},R_{c}^{c}$), of the ΔG(N,〈R〉)-curve for the case when maximum and minimum coincide.

Generally, *Z* obeys the relation Z<−1. Its absolute value increases rapidly with increasing average cluster size. The value of the parameter ω is specified later. The quantity $\Phi^{\left(\varepsilon \right)}$ denotes the total energy of elastic deformations, due to the formation of a cluster of size *R* or volume $V_{\alpha }$. The elastic energy is assumed here to be caused by matrix cluster interactions. Cluster–cluster interactions, as first modeled by Kawasaki and Enomoto [59,60], are not included here. For their description, an additional term depending on the number of clusters has to be introduced.

If elastic stresses are absent ($\Phi^{\left(\varepsilon \right)}=0$), or proportional to the volume of the cluster (i.e., $\Phi^{\left(\varepsilon \right)}=\varepsilon V_{\alpha }$ as assumed in the first analysis of Lifshitz and Slezov devoted to the effect of elastic stresses on coarsening [61]), then elastic terms vanish from the equations and we obtain:

(56) $$\frac{d\langle R\rangle }{dt}=\omega \frac{D\sigma c}{c_{\alpha }^{2}k_{B}T}\frac{1}{\langle R\rangle l}\left(1+\frac{1}{Z}\right)\;,$$

(57) $$\frac{d\ln N}{dt}=-3\left(1+\frac{1}{Z}\right)\frac{d\ln\langle R\rangle }{dt}\;,$$

or, equivalently,

(58) $$\frac{d}{dt}\ln\left(N{\langle R\rangle }^{3}\right)=-\frac{1}{Z}\frac{d}{dt}\left[\ln{\langle R\rangle }^{3}\right]\;.$$

These equations allow us to describe the whole course of coarsening indicated by a dashed line in Figure 8, including its initial stages.

At the state ($N_{c},R_{c}^{c}$), giving the lowest value of the average cluster size and the highest value of the number of clusters at which coarsening may start, we have Z=−1. In the asymptotic stage of coarsening, the quantity *Z* tends to minus infinity and the well-known expressions for the description of the coarsening, as derived in the LSW−theory, are obtained as special cases. The present relations allow us, in addition to the asymptotic stage of coarsening, also to describe the initial stage of this process when the total amount of the newly evolving phase is still increasing with time.

In the description of segregation processes in solutions at non−zero input fluxes of segregating particles, we assume that elastic stresses are negligible. Anyway, we briefly also discuss such an effect here in order to show that it is the behavior of the quantity *Z* that determines the differences in coarsening behavior, as compared to the cases described by the LSW−theory. In the subsequent analysis, we illustrate the method of derivation of these relations and concentrate on the question of how they have to be generalized in order to describe coarsening at non−zero input fluxes of segregating particles.

#### 3.3.2. Derivation of the Kinetic Equations Modeling Coarsening in a Closed System

As a first step in the derivation of the basic relations, we realize that Equations (3) and (4) can be rewritten as:

(59) $$\frac{dR}{dt}=-\frac{Dc}{c_{\alpha }^{2}k_{B}T}\frac{1}{4\pi R^{2}l}\left(\frac{\partial \Delta G}{\partial R}\right)\;,$$

utilizing Equation (55), again, i.e., taking l=R for diffusion−limited growth and $l=l_{0}$ for kinetic−limited growth. Indeed, a substitution of Equation (23) into Equation (59) utilizing Equations (17) and (18) immediately results in Equations (3) and (4).

Considering an ensemble of *N* identical clusters with the same size, *R*, for each of them we can write an expression of the form of Equation (59). The parameter *R* we identify with the average size, 〈R〉, of the real ensemble of clusters. Taking the sum over all clusters we obtain then:

(60) $$N\frac{d\langle R\rangle }{dt}=-\frac{Dc}{c_{\alpha }^{2}k_{B}T}\frac{1}{4\pi {\langle R\rangle }^{2}l}\left(\frac{\partial \Delta G(N,\langle R\rangle )}{\partial \langle R\rangle }\right)\;.$$

Here ΔG has to be formulated as a function of *N* and 〈R〉, i.e., ΔG=ΔG(N,〈R〉).

In line with the adapted here alternative theoretical description of coarsening as the motion of the clusters along the “valley” of the thermodynamic potential, ΔG(N,〈R〉), we set:

(61) $$\left(\frac{\partial \Delta G(N,\langle R\rangle )}{\partial \langle R\rangle }\right)=\omega \frac{d\Delta G(N,\langle R\rangle )}{dN}\frac{dN}{d\langle R\rangle }\;.$$

Here ω is a numerical correction factor entering also the final relations, Equations (53) and (56). It is determined later, when comparing our results with the predictions of the LSW−theory.

In order to complete the analysis, we have to determine how ΔG(N,〈R〉) depends on the number of clusters along the considered evolution path and how the number of clusters and their average size are correlated. For that purpose, we employ Gibbs’ fundamental theory of surface phenomena [62] also utilizing methods developed by Rusanov [63,64] and advanced further by some of us in [8]. Note that similar results could also be obtained in terms of the generalized Gibbs’ approach as described by some of us in [9].

In order to proceed in this direction, first we consider the general case that, in a multi-component ambient phase with the molar fractions, $x_{j\beta }$, of the j=1,2,…,k different components clusters of a new phase are formed with a given composition described by the set of molar fractions, $x_{j\alpha }$. The quantity $n_{\alpha }$ denotes the total number of particles in a cluster with a volume density of the particles equal to $c_{\alpha }$. Similarly to Equations (17) and (18), we may write:

(62) $$n_{\alpha }=\frac{4\pi }{3}c_{\alpha }R^{3}=c_{\alpha }V_{\alpha }\;.$$

We describe the composition in the initial state of the ambient phase by the set of molar fractions of the different components, $x_{j}$. The process is assumed to take place at a given temperature and pressure. The change of the Gibbs free energy caused by the evolution of one cluster can be then expressed in a good approximation as [8,9,32,42,43,44,65,66]:

(63) $$\begin{array}{cc} \Delta G(R) & =-\sum_{j=1}^{k}n_{j\alpha }\left[\mu_{j\beta }\left(p,T,\{x_{j\beta }\}\right)-\mu_{j\alpha }\left(p,T,\{x_{j\alpha }\}\right)\right]\\ & +\sum_{j=1}^{k}n_{j}\left[\mu_{j\beta }\left(p,T,\{x_{j\beta }\}\right)-\mu_{j}\left(p,T,\{x_{j}\}\right)\right]+4\pi R^{2}\sigma \end{array}$$

or

(64) $$\begin{array}{cc} \Delta G(R) & =-\frac{4\pi }{3}c_{\alpha }R^{3}\sum_{j=1}^{k}x_{j\alpha }\left[\mu_{j\beta }\left(p,T,\{x_{j\beta }\}\right)-\mu_{j\alpha }\left(p,T,\{x_{j\alpha }\}\right)\right]\\ & +\sum_{j=1}^{k}n_{j}\left[\mu_{j\beta }\left(p,T,\{x_{j\beta }\}\right)-\mu_{j}\left(p,T,\{x_{j}\}\right)\right]+4\pi R^{2}\sigma \;. \end{array}$$

Equations (63) and (64) describe the change of the Gibbs free energy in dependence on cluster size, accounting for changes of the state of the ambient phase caused by the evolution of the cluster.

If *N* clusters of the same size formed in a given ambient phase, then we obtain:

(65) $$\begin{array}{cc} \Delta G(R,N) & =-\frac{4\pi }{3}c_{\alpha }R^{3}N\sum_{j=1}^{k}x_{j\alpha }\left[\mu_{j\beta }\left(p,T,\{x_{j\beta }\}\right)-\mu_{j\alpha }\left(p,T,\{x_{j\alpha }\}\right)\right]\\ & +\sum_{j=1}^{k}n_{j}\left[\mu_{j\beta }\left(p,T,\{x_{j\beta }\}\right)-\mu_{j}\left(p,T,\{x_{j}\}\right)\right]+4\pi R^{2}N\sigma \;. \end{array}$$

The extremes of ΔG(R,N) for constant numbers of clusters are given by:

(66) $${\left.\frac{\partial \Delta G(R,N)}{\partial R}\right|}_{N}=-4\pi c_{\alpha }R^{2}N\left\{\sum_{j=1}^{k}x_{j\alpha }\left[\mu_{j\beta }\left(p,T,\{x_{j\beta }\}\right)-\mu_{j\alpha }\left(p,T,\{x_{j\alpha }\}\right)\right]-\frac{2\sigma }{c_{\alpha }R}\right\}\;.$$

Terms, containing derivatives of the chemical potential $\mu_{j\beta }$ with respect to *R*, vanish due to the Gibbs–Duhem relation, $\sum_{j}n_{j\beta }d\mu_{j\beta }=0$.

The second-order derivative with respect to the cluster radius can be written as:

(67) $${\left.\frac{\partial^{2}\Delta G\left(R,N\right)}{\partial R^{2}}\right|}_{N}=-4\pi c_{\alpha }R^{2}N\left\{{\left.\frac{\partial }{\partial R}\left(\sum_{j=1}^{k}x_{j\alpha }\mu_{j\beta }\left(p,T,\{x_{j\beta }\}\right)\right)\right|}_{N}+\frac{2\sigma }{c_{\alpha }R^{2}}\right\}$$

or

(68) $${\left.\frac{\partial^{2}\Delta G\left(R,N\right)}{\partial R^{2}}\right|}_{N}=-8\pi \sigma RN\left(1+Z\right)$$

with

(69) $$Z=\frac{c_{\alpha }R^{2}}{2\sigma }{\left.\frac{\partial }{\partial R}\left(\sum_{j=1}^{k}x_{j\alpha }\mu_{j\beta }\left(p,T,\{x_{j\beta }\}\right)\right)\right|}_{N}\;.$$

In the model advanced here, coarsening is supposed to proceed as the evolution along the valley of the relevant for the given conditions thermodynamic potential difference. The trajectory of evolution is determined by (∂ΔG/∂R)=0 and $(\partial^{2}\Delta G/\partial R^{2})>0$ keeping the number of clusters fixed in performing the derivatives. Accounting for these conditions and Equation (68), the inequality Z<−1 had to be generally fulfilled for the evolution path considered.

Depletion effects (changes of the state of the ambient phase) are determined by the total volume of the newly evolving phase proportional to $\theta =R^{3}N$. For this reason, we may rewrite the expression for *Z* as:

(70) $$Z=\frac{c_{\alpha }R^{2}}{2\sigma }\frac{\partial }{\partial \theta }\left(\sum_{j=1}^{k}x_{j\alpha }\mu_{j\beta }\left(p,T,\{x_{j\beta }\}\right)\right)3NR^{2}\;.$$

As noted, *Z* had negative values along the valley of the thermodynamic potential. It is equal to minus one at the state specified in Figure 8 by ($N_{c},R_{c}^{c}$), since at this point the second-order derivative of *G*, with respect to *R*, is also equal to zero. The analysis of coarsening in different systems always led to the same result, i.e., that its absolute value increases in the evolution along this path, provided additional factors like elastic stresses are absent. This conclusion is reconfirmed when we consider the consequences for the particular model analyzed in detail in this paper.

Taking the derivatives of ΔG(R,N) with respect to *N* for values of *R* corresponding to the extremes of ΔG(R,N), we obtain:

(71) $$\frac{\partial \Delta G(R,N)}{\partial N}=-\frac{4\pi }{3}c_{\alpha }R^{3}\sum_{j=1}^{k}x_{j\alpha }\left[\mu_{j\beta }\left(p,T,\{x_{j\beta }\}\right)-\mu_{j\alpha }\left(p,T,\{x_{j\alpha }\}\right)\right]+4\pi R^{2}\sigma$$

and, accounting for Equation (66):

(72) $${\left.\frac{\partial \Delta G(R,N)}{\partial N}\right|}_{\mathrm{Extrema}}=\frac{4\pi }{3}\sigma R^{2}\;.$$

Finally, the Gibbs–Thomson equation (cf. Equation (66)):

(73) $$\sum_{j=1}^{k}x_{j\alpha }\left[\mu_{j\beta }\left(p,T,\{x_{j\beta }\}\right)-\mu_{j\alpha }\left(p,T,\{x_{j\alpha }\}\right)\right]=\frac{2\sigma }{c_{\alpha }R}$$

supplies us with a general relation connecting *N* and *R*. Taking the derivative of Equation (73) with respect to *R* and accounting for the dependence N=N(R), we obtain:

(74) $$\frac{d}{dR}\left(\sum_{j=1}^{k}x_{j\alpha }\mu_{j\beta }\left(p,T,\{x_{j\beta }\}\right)\right)=-\frac{2\sigma }{c_{\alpha }R^{2}}\;,$$

respectively,

(75) $$Z\frac{d\theta }{dR}=-3NR^{2}$$

or, finally,

(76) $$\frac{dN}{dR}=-\frac{3N}{R}\left(\frac{1+Z}{Z}\right)\;.$$

Identifying *R* with 〈R〉, we directly obtain from Equations (60), (61), (72), and (76) the first of the Equations (56)–(58). Equation (76) immediately yields:

(77) $$\frac{d\ln N}{dt}=-3\frac{d\ln R}{dt}\left(1+\frac{1}{Z}\right)\;.$$

It can be easily transformed into Equation (57) employing the relation:

(78) $$3\frac{d\ln R}{dt}=\frac{d\ln{\left(R\right)}^{3}}{dt}\;.$$

The equations derived here allow us to describe coarsening in the whole range of the process, describing it in terms of the motion along the valley of the thermodynamic potential surface. In order to arrive at the same results as the theory of Lifshitz and Slezov supplies for the asymptotic region of coarsening, we have to set the numerical parameter ω equal to:

(79) $$\omega =\left\{\begin{array}{cc} \frac{8}{27} & \mathrm{for diffusion–limited growth}\\ \; & \\ \frac{1}{2} & \mathrm{for kinetic–limited growth} \end{array}\right.\;.$$

In this way, the derivation of the basic equations describing coarsening in closed systems in an alternative way, as compared to the method developed by Lifshitz and Slezov, is completed. The results demonstrate the principal validity of the model employed and the power of thermodynamic methods in the analysis of the kinetics of phase transformation processes. Going beyond the LSW−theory, the method allows the theoretical description not only of the asymptotic, but also of the initial, stages of coarsening. In addition, the method provides the possibility to account for the effect of additional factors, like elastic stresses, on coarsening as it is demonstrated in Section 3.3.3, giving the background for the subsequent application of the method to coarsening in open systems.

#### 3.3.3. Account of Additional Factors like Elastic Stresses on Coarsening

Accounting for elastic stresses, we have to add the energy of elastic deformations, $\Phi^{\left(\varepsilon \right)}$, caused by the formation of a cluster to the Gibbs free energy [27,28]. In addition, variations of the surface tension, due to elastic stresses, as described in [67], have to be accounted for, in general. The latter effect was neglected in the present treatment.

Incorporating elastic stress effects caused by the evolution of the aggregates into the description, the change of the Gibbs free energy is given, similarly to Equation (65), as:

(80) $$\begin{array}{cc} \Delta G(R,N) & =-\frac{4\pi }{3}c_{\alpha }R^{3}N\sum_{j=1}^{k}x_{j\alpha }\left[\mu_{j\beta }\left(p,T,\{x_{j\beta }\}\right)-\mu_{j\alpha }\left(p,T,\{x_{j\alpha }\}\right)\right]\\ & +\sum_{j=1}^{k}n_{j}\left[\mu_{j\beta }\left(p,T,\{x_{j\beta }\}\right)-\mu_{j}\left(p,T,\{x_{j}\}\right)\right]+4\pi R^{2}N\sigma +N\Phi^{\left(\varepsilon \right)}\;. \end{array}$$

The extreme values of ΔG(R,N) for constant numbers of clusters are given by:

(81) $$\begin{array}{cc} \; & {\left.\frac{\partial \Delta G(R,N)}{\partial R}\right|}_{N}=-4\pi c_{\alpha }R^{2}N\times \\ & \times \left\{\sum_{j=1}^{k}x_{j\alpha }\left[\mu_{j\beta }\left(p,T,\{x_{j\beta }\}\right)-\mu_{j\alpha }\left(p,T,\{x_{j\alpha }\}\right)\right]-\frac{2\sigma }{c_{\alpha }R}-\frac{1}{4\pi c_{\alpha }R^{2}}\frac{\partial \Phi^{\left(\varepsilon \right)}}{\partial R}\right\}\;. \end{array}$$

Terms, with derivatives of the chemical potentials, $\mu_{j\beta }$, with respect to *R*, vanish, again, due to the Gibbs–Duhem relation, $\sum_{j}n_{j\beta }d\mu_{j\beta }=0$. The second-order derivative, with respect to the cluster radius, can be written as:

(82) $${\left.\frac{\partial^{2}\Delta G\left(R,N\right)}{\partial R^{2}}\right|}_{N}=-4\pi c_{\alpha }R^{2}N\left\{{\left.\frac{\partial }{\partial R}\left(\sum_{j=1}^{k}x_{j\alpha }\mu_{j\beta }\left(p,T,\{x_{j\beta }\}\right)\right)\right|}_{N}+\frac{2\sigma }{c_{\alpha }R^{2}}-\frac{1}{c_{\alpha }}\frac{\partial^{2}\Phi^{\left(\varepsilon \right)}}{\partial R\partial V_{\alpha }}\right\}$$

or

(83) $$\;\;\;\;\;\;\;\;\;\;\;{\left.\frac{\partial^{2}\Delta G\left(R,N\right)}{\partial R^{2}}\right|}_{N}=-8\pi \sigma RN\left(1+Z-\frac{R^{2}}{2\sigma }\frac{\partial^{2}\Phi^{\left(\varepsilon \right)}}{\partial R\partial V_{\alpha }}\right)\;.$$

With $V_{\alpha }$, we denote here the volume of the cluster, again.

Taking the derivatives of ΔG(R,N), with respect to *N*, for values of *R*, corresponding to the extremes of ΔG(R,N), we obtain:

(84) $$\frac{\partial \Delta G(R,N)}{\partial N}=-\frac{4\pi }{3}c_{\alpha }R^{3}\sum_{j=1}^{k}x_{j\alpha }\left[\mu_{j\beta }\left(p,T,\{x_{j\beta }\}\right)-\mu_{j\alpha }\left(p,T,\{x_{j\alpha }\}\right)\right]+4\pi R^{2}\sigma +\Phi^{\left(\varepsilon \right)}$$

and, accounting for Equation (81):

(85) $${\left.\frac{\partial \Delta G(R,N)}{\partial N}\right|}_{\mathrm{Extrema}}=\frac{4\pi }{3}\sigma R^{2}+\Phi^{\left(\varepsilon \right)}-V_{\alpha }\frac{\partial \Phi^{\left(\varepsilon \right)}}{\partial V_{\alpha }}\;.$$

Consequently, as far as $\Phi^{\left(\varepsilon \right)}=\varepsilon V_{\alpha }$ holds, the coarsening kinetics is not modified qualitatively by elastic stresses. However, if elastic stresses grow more rapidly than linear with the volume of the cluster, then, in the course of time, a mono-disperse cluster size distribution is established in Ostwald ripening, as demonstrated in [21,27,28,50,51,52].

Finally, the Gibbs–Thomson equation (cf. Equation (81)):

(86) $$\sum_{j=1}^{k}x_{j\alpha }\left[\mu_{j\beta }\left(p,T,\{x_{j\beta }\}\right)-\mu_{j\alpha }\left(p,T,\{x_{j\alpha }\}\right)\right]=\frac{2\sigma }{c_{\alpha }R}+\frac{1}{c_{\alpha }}\frac{\partial \Phi^{\left(\varepsilon \right)}}{\partial V_{\alpha }}$$

supplies as with a general relation connecting *N* and *R*. Taking the derivative of Equation (86), with respect to *R*, and accounting for the dependence N=N(R), we obtain:

(87) $$\frac{dN}{dR}=-\frac{3N}{R}\left(\frac{1+Z-\frac{R^{2}}{2\sigma }\frac{\partial^{2}\Phi^{\left(\varepsilon \right)}}{\partial R\partial V_{\alpha }}}{Z}\right)\;.$$

Identifying *R* with 〈R〉 and $V_{\alpha }$ with $\langle V_{\alpha }\rangle$, we directly obtain, from Equations (60), (61), (78), (85), and (87), the basic relations, Equations (53) and (54). Consequently, knowing the dependence of the energy of elastic deformations on the size of an aggregate, immediately their effect on the time evolution of the number of clusters and their average sizes in coarsening can be determined.

For particular models, a more detailed description of the kinetics of coarsening under the influence of elastic stresses and in porous systems, including the determination of the dependence of the cluster size distribution on time, was performed by one of us in cooperation with J. Möller and V. V. Slezov in [68,69,70,71]. It is a much more complex task, as compared with the approach discussed here and resulting into the relatively simple relations, Equations (53) and (54).

#### 3.3.4. Application to Coarsening at Non-Zero Input Fluxes of Segregating Particles

The method outlined in Section 3.3.1, Section 3.3.2 and Section 3.3.3, we now employ to describe coarsening at non-zero input fluxes of segregating particles. We start with the consideration that, at any moment of time, the change of the Gibbs free energy, in dependence on the number of clusters and their average sizes, is given by curves with a shape as illustrated in Figure 8. However, since the total number of segregating particles changes with time, the curves and, in particular, the valley of the thermodynamic potential surface changes with time as well. This effect we will now incorporate into the description of coarsening for a particular model commonly employed in the description of coarsening in solid solutions in closed systems and already utilized in Section 3.2, in performing the numerical computations for coarsening at non-zero input fluxes of segregating particles.

As a particular case in this approach, described in Section 3.3.1, Section 3.3.2 and Section 3.3.3, we consider here segregation in a binary solution containing initially $n_{1}$ and $n_{2}$ particles in a given volume. Component one segregates into clusters consisting exclusively of this component with a number of particles denoted as $n_{\alpha }$ occupying a volume, $V_{\alpha }=\left(4\pi /3\right)R^{3}$. In the course of nucleation and growth the number of particles of component one in the solution changes. Its current value is denoted by $n_{1\beta }$. The chemical potentials per particle we denote by $\mu_{1\alpha }$ (cluster phase), $\mu_{i}$ (initial state in the ambient phase), and $\mu_{i\beta }$ (accounting for its change caused by segregation processes). For any moment of time, *t*, respectively, any value, $n_{1}\left(t\right)$, of the total number of particles of component one in the system, we get, as a special case of Equation (65), the following result for the change of the Gibbs free energy caused by the formation of *N* clusters of the same sizes [8,50,63]:

(88) $$\Delta G=n_{\alpha }N\left(\mu_{1\alpha }-\mu_{1\beta }\right)+\sum_{j=1}^{2}n_{j}\left(\mu_{j\beta }-\mu_{j}\right)+4\pi R^{2}N\sigma \;.$$

Assuming that the ambient phase can be described as a perfect solution:

(89) $$\mu_{j}=\mu_{j}^{*}+k_{B}T\ln x_{j}\;,\;x_{1}=\frac{n_{1}}{n_{1}+n_{2}}\;,\;x_{2}=1-x_{1}\;,$$

we arrive at:

(90) $$\Delta G=-n_{\alpha }Nk_{B}T\ln\left(\frac{c_{\beta }}{c_{eq}}\right)+n_{1}k_{B}T\ln\left(\frac{x_{\beta }}{x}\right)+n_{2}k_{B}T\ln\left(\frac{1-x_{\beta }}{1-x}\right)+4\pi R^{2}N\sigma$$

with

(91) $$c=\frac{n_{1}}{V}\;,\;c_{\beta }=c\frac{1-\frac{Nn_{\alpha }}{n_{1}}}{1-\frac{NV_{\alpha }}{V}}\;,\;n_{\alpha }=c_{\alpha }V_{\alpha }\;,$$

respectively,

(92) $$\begin{array}{cc} \frac{\Delta G(N,R;t)}{k_{B}T} & =-Nn_{\alpha }\ln\left(\frac{c\left(t\right)\left(1-\frac{Nn_{\alpha }}{n_{1}\left(t\right)}\right)}{c_{eq}\left(1-\frac{NV_{\alpha }}{V}\right)}\right)+\frac{4\pi R^{2}N\sigma }{k_{B}T}\\ \; & +n_{1}\ln\left(\frac{x_{\beta }}{x}\right)+n_{2}\ln\left(\frac{1-x_{\beta }}{1-x}\right)\;. \end{array}$$

Accounting for non-zero input fluxes of segregating particles, the total number of segregating particles in the system, $n_{1}\left(t\right)$, and their concentration, $c\left(t\right)=n_{1}/V$, are given by Equation (25).

Performing the thermodynamic analysis for a given moment of time, or a given value of *c*, instead of Equations (66), (67), (68), (70) and (72), we arrive at:

(93) $${\left.\frac{\partial }{\partial R}\frac{\Delta G(R,N;t)}{k_{B}T}\right|}_{N}=-\frac{4\pi c_{\alpha }R^{2}N}{k_{B}T}\left\{k_{B}T\ln\left(\frac{c\left(t\right)\left(1-\frac{Nn_{\alpha }}{n_{1}\left(t\right)}\right)}{c_{eq}\left(1-\frac{NV_{\alpha }}{V}\right)}\right)-\frac{2\sigma }{c_{\alpha }R}\right\}\;,$$

(94) $${\left.\frac{\partial^{2}}{\partial R^{2}}\frac{\Delta G(R,N;t)}{k_{B}T}\right|}_{N}=-\frac{4\pi c_{\alpha }R^{2}N}{k_{B}T}\left\{{\left.\frac{\partial }{\partial R}\left(k_{B}T\ln\left(\frac{c\left(t\right)\left(1-\frac{Nn_{\alpha }}{n_{1}\left(t\right)}\right)}{c_{eq}\left(1-\frac{NV_{\alpha }}{V}\right)}\right)\right)\right|}_{N}+\frac{2\sigma }{c_{\alpha }R^{2}}\right\}$$

or

(95) $${\left.\frac{\partial^{2}\Delta G\left(R,N\right)}{\partial R^{2}}\right|}_{N}=-8\pi \sigma RN\left(1+Z\right)\;,$$

(96) $$Z=\frac{c_{\alpha }R^{2}}{2\sigma }{\left.\frac{\partial }{\partial R}\left(k_{B}T\ln\left(\frac{c\left(t\right)\left(1-\frac{Nn_{\alpha }}{n_{1}\left(t\right)}\right)}{c_{eq}\left(1-\frac{NV_{\alpha }}{V}\right)}\right)\right)\right|}_{N}\;,$$

respectively,

(97) $$Z=\frac{2\pi k_{B}Tc_{\alpha }R^{4}N}{\sigma }\frac{\partial }{\partial \theta^{\prime }}\left(\ln\left(\frac{c\left(t\right)\left(1-\frac{c_{\alpha }\theta^{\prime }}{n_{1}\left(t\right)}\right)}{c_{eq}\left(1-\frac{\theta^{\prime }}{V}\right)}\right)\right)\;,\;\theta^{\prime }=\frac{4\pi }{3}NR^{3}\;.$$

The quantity *Z* can be expressed in the considered case as:

(98) $$Z=-\frac{2\pi k_{B}Tc_{\alpha }R^{4}N}{V\sigma }\frac{\left(\frac{c_{\alpha }}{c(t)}-1\right)}{\left(1-\frac{c_{\alpha }}{c(t)}\frac{\theta^{\prime }}{V}\right)\left(1-\frac{\theta^{\prime }}{V}\right)}\;.$$

The parameter $\theta^{\prime }$ is the total volume of the segregating phase and $c_{\alpha }\theta^{\prime }$ the total number of particles segregated into clusters. Both quantities have to be smaller compared to the volume of the ambient solution and the number of segregating particles originally dissolved in it, i.e., the inequalities $c_{\alpha }\theta^{\prime }<cV$ and $\theta^{\prime }<V$ have to be fulfilled. In addition, the inequality $c_{\alpha }\ll c$ has to be fulfilled. Physically reasonable results of the solution of the differential equations employed in the analysis have to obey these conditions.

Equations (56)–(58) remain unchanged. However, the concentration, *c*, in the pre-factor of Equation (56) has to be identified, now, with the current concentration in the ambient phase, $c_{\beta }$. The main modifications of the coarsening behavior are determined by the behavior of the quantity *Z* in dependence on time. Going over to the dimensionless time scale (Equation (19)), we obtain the basic kinetic equations for the description of coarsening in open systems of the considered type in the form:

(99) $$\frac{d\langle R\rangle }{dt^{\prime }}=\omega \frac{\sigma (c_{\beta }/c_{eq})}{4\pi c_{\alpha }^{2}k_{B}TR_{1}}\frac{1}{\langle R\rangle l}\left(1+\frac{1}{Z}\right)\;,$$

(100) $$\frac{d\ln N}{dt^{\prime }}=-3\left(1+\frac{1}{Z}\right)\frac{d\ln\langle R\rangle }{dt}\;.$$

**Figure 9:**
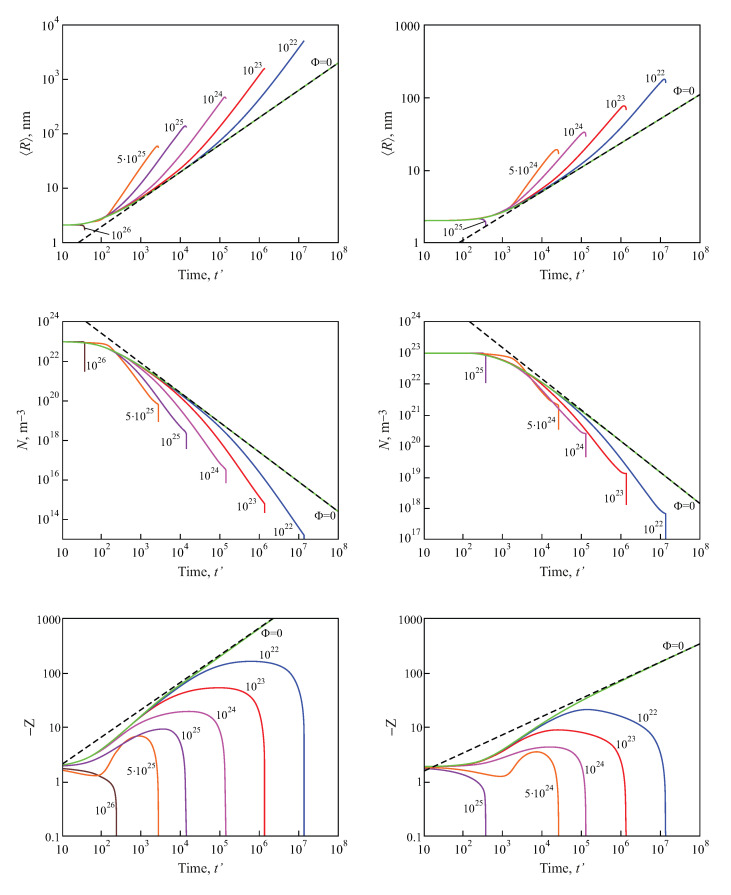
Predictions of Equations (99) and (100) modeling the evolution in time of the average size and the number of clusters in coarsening in an open system in a volume of the solution equal to $1\;\;m^{3}$ for both kinetic−limited (**left column**) and diffusion−limited (**right column**) growth modes and different input fluxes of segregating particles, as indicated in the figures.

Numerical solutions of these equations, modeling the average size and the number of clusters in a volume of the solution equal to $1\;\;m^{3}$, are shown in Figure 9, for both diffusion− and kinetic−limited growth. The initial conditions were set equal to: 〈R(0)〉=2nm, $N\left(0\right)=10^{23}\;\;m^{-3}$, $c\left(0\right)=4c_{eq}$. The dashed curves reproduce the kinetics of coarsening in closed systems, resulting in $\langle R\rangle \propto t^{\left(1/2\right)}$, $N\propto 1/t^{3/2}$ for kinetic−limited growth and $\langle R\rangle \propto t^{\left(1/3\right)}$, N∝1/t for diffusion−limited growth. The full curves provide the dependencies of the number of clusters and their average sizes for different input fluxes of the segregating particles. For any chosen value of time, the average cluster sizes were larger and the numbers of clusters were smaller, as compared to coarsening in closed systems. Note also that the curves refer to physically reasonable situations only, as far as the condition Z<−1 (or −Z>1) are fulfilled.

#### 3.3.5. Possible Further Applications of the Method: General Theoretical Approach to the Description of Coarsening in Open Systems and at Time-Dependent Boundary Conditions

Having completed the analysis for the particular case of coarsening at constant input fluxes of segregating particles, we would like to underline immediately that the method is generally applicable. It can be utilized similarly for cases when the input fluxes of segregating particles are changing with time. More generally, it allows the description of coarsening in open systems or in situations where the boundary conditions, like pressure and temperature, are changing with time.

For any given moment of time, one has to formulate expressions for the characteristic thermodynamic potential, like ΔG(〈R〉,N) shown in Figure 8. For that moment, one can obtain the respective results for the change of the average cluster size and the number of clusters. For open systems and/or systems in which the boundary conditions are changing with time, the potential surface changes also with time. Consequently, the respective expressions for the growth kinetics also vary with time, caused by the time-dependence of some parameters in the basic kinetic equations as in the particular case here analyzed. The model is generally applicable and may be employed to interpret existing or new experimental data on the evolution of cluster size distributions under mentioned conditions.

Having at one’s disposal this method for the determination of the average cluster size and the number of clusters, conditions can be tested theoretically at which desired cluster size distributions (number of clusters and average size) develop most appropriate for particular technological applications. This problem is widely discussed in the literature. The method developed in the present paper supplies us with a new tool to solve it.

## 4. Summary of Results and Discussion

As shown in the present analysis, processes of segregation in a solid or liquid solution at a constant rate of supply of segregating monomers can be divided into several distinct stages, similar to the kinetics of nucleation-growth processes in segregation in solutions in closed systems (cf. Figure 1 and Figure 8). Hereby, the general scenario of the evolution does not depend on the particular mechanism of growth of the clusters (e.g., kinetically− or diffusion−limited growth, as considered here).

In a first stage of the process, the relative supersaturation increases monotonically with time, due to the supply of monomers to the system (Figure 3). After the initiation of nucleation, which occurs with perceptible intensity after some critical value of the supersaturation is reached, an ensemble of growing (supercritical) clusters is formed and, initially, the total number of clusters in the system increases (Figure 5). In this second interval of intensive nucleation, the critical cluster radii are small and almost all supercritical clusters are growing rapidly, while, at the same time, new supercritical clusters are formed.

The total number of clusters formed increases with an increase of the input fluxes of segregating particles (Figure 7). As the result of both processes the degree of supersaturation (excess of monomers) achieved initially is reduced (Figure 3). The decrease of relative supersaturation leads to an increase of the critical cluster radius (Figure 4). As a result, in a third stage of the process, some of the clusters become sub-critical and are dissolved. As a consequence, the total number of clusters decreases (see Figure 5). Finally, the system approaches a fourth steady state where the changes in the relative supersaturation become small and a balance between the input fluxes and the consumption of monomers by the growing clusters is established, supplemented by a further decrease in the number of clusters and an increase in their average sizes.

In the present analysis, processes of segregation in open systems were analyzed by numerical computations solving the appropriate set of kinetic equations of CNT. The numerical results were confirmed by analytical methods directed to the determination of the dependence of the number of clusters on the rate of input fluxes of segregating particles. The results are shown to be in agreement with experimental data. In particular, a theory of coarsening in open systems was developed. It was demonstrated that the results obtained for the dependence of the number of clusters and their average sizes on time are different as compared to the predictions of the Lifshitz–Slezov–Wagner theory, which describes the kinetics of Ostwald ripening in closed systems. At zero-input fluxes of segregating particles, the predictions of our approach coincide with the results of the LSW−theory.

**Figure 10:**
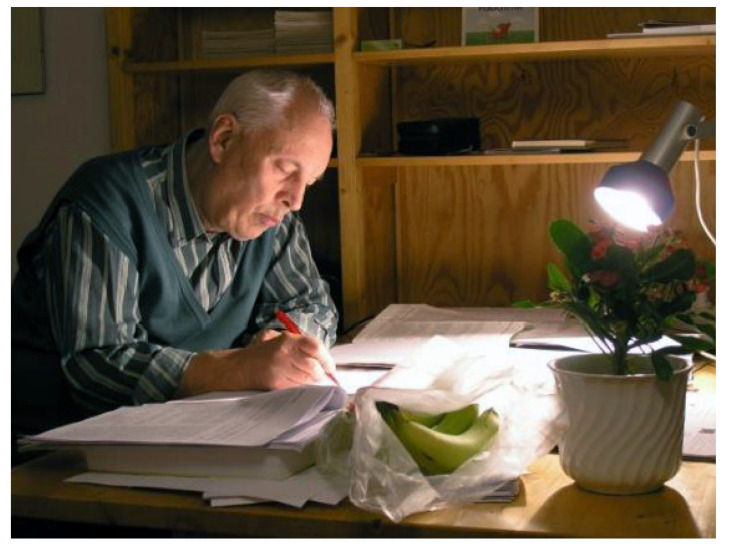
The present paper is devoted to our teacher, colleague, and friend, the Corresponding Member of the Ukrainian Academy of Sciences, Vitali V. Slezov (9 March 1930–30 October 2013).

We would like to underline here that the method advanced by us is applicable generally as a tool for the analysis of coarsening at changing boundary conditions and coarsening in open systems going beyond the theory developed first by Lifshitz and Slezov. In this respect, we consider the present paper as a continuation and extension of their work and would like to devote the present paper to our teacher, colleague, and friend, Vitali V. Slezov (cf. Figure 10). We discussed the necessity of the presented extension of his work with him long ago but could successfully complete it, as we hope we have, only now.

## Data Availability

Not applicable.
